# Internal biases are linked to disrupted cue combination in children and adults

**DOI:** 10.1167/jov.22.12.14

**Published:** 2022-11-15

**Authors:** James Negen, Heather Slater, Laura-Ashleigh Bird, Marko Nardini

**Affiliations:** 1 School of Psychology, Liverpool John Moores University, Liverpool, UK; 2 Department of Psychology, Durham University, Durham, UK; 3 Institute of Cognitive Neuroscience, University College London, London, UK; 4 Department of Psychology, Durham University, Durham, UK; 5 Department of Psychology, Durham University, Durham, UK; 6 MRC Cognition and Brain Sciences Unit, University of Cambridge, Cambridge, UK

**Keywords:** bias, cue combination, Bayesian models

## Abstract

Cue combination describes the use of two sensory cues together to increase perceptual precision. Internal relative bias describes a situation in which two cues to the same state of the world are perceived as signaling different states of the world on average. Current theory and evidence have difficulty accounting for many instances where cue combination is absent, such as in children under 10 years old, and in a variety of tasks. Here we show that internal relative biases between cues could be a key explanatory factor. Experiment 1, studying children's three-dimensional (slant) perception via disparity and texture, found a negative cross-sectional correlation between internal relative bias and cue combination behavior in 7- to 10-year-olds. Strikingly, children who had below-median levels of internal relative bias were able to combine cues, unlike the typical result for that age range. Experiment 2, studying adults’ visual-auditory localization, found that cue combination behavior increased after an intervention designed to decrease internal relative bias. We interpret this as strong but preliminary evidence that internal relative bias can disrupt cue combination behavior. This provides a plausible mechanism to explain why children under 10 generally do not combine cues and why the audiovisual cue combination is so inconsistent in adults. Moving forward, we suggest that researchers who fail to find an expected cue combination effect should further investigate the possibility of issues with internal relative bias. Decreasing internal relative bias may also be an important goal for rehabilitation and sensory substitution or augmentation approaches to promoting efficient multisensory perception.

## Introduction

Human perception uses a variety of methods to make efficient use of the sensory signals available to make estimates about the physical world (Maloney & Mamassian, 2009). One of these methods, cue combination, is combining (averaging) multiple sensory cues to a single object or property in order to make a judgment. An advantage of cue combination is that it reduces random noise, making the combined estimate more precise than either single-cue estimate. For example, when adults are given a noisy visual cue and a noisy audio cue to horizontal location together, they can be more precise than with either cue alone (Alais & Burr, 2004). Cue combination has been modeled as statistical inference: With multiple cues available, the response is equal to the maximum likelihood estimate (Ernst & Banks, 2002), which is also the Bayesian maximum a posteriori under certain conditions (Ernst, 2006). The ability to combine cues in this way allows perception to efficiently integrate all of the available information. Cue combination seems to be a common process in both multisensory and unisensory processing (Ma, 2010; Pouget, Beck, Ma, & Latham, 2013; Trommershäuser, Körding, & Landy, 2012), with a basis in the early sensory processing of the brain (Ban, Preston, Meeson, & Welchman, 2012; Gu Angelaki, & DeAngelis, 2008) and also across a whole hierarchy of areas mediating perception and decision-making (Rohe & Noppeney, 2015; Rohe & Noppeney, 2016). To understand perception and its core principles, we need to understand cue combination in detail.

One major issue with theory relating to cue combination is that we have limited ability to predict when it will or will not occur, restricting the explanatory power of this framework. Although cue combination effects (i.e., greater precision with multiple cues than any alone) have been reported in a wide variety of settings (Ma, 2010; Pouget et al., 2013; Trommershäuser et al., 2012), numerous studies have failed to find cue combination effects (Rahnev & Denison, 2018). For example, most studies have found that children below around 10 years old do not show the predicted precision benefits, whether in multisensory tasks (Adams, 2016; Burr & Gori, 2011; Gori, Del Viva, Sandini, & Burr, 2008; Jovanovic & Drewing, 2014; Nardini, Begus, & Mareschal, 2013; Nardini, Jones, Bedford, & Braddick, 2008; Petrini, Remark, Smith, & Nardini, 2014) or in unisensory tasks (Dekker, Ban, van der Velde, Sereno, Welchman, & Nardini, 2015; Nardini, Bedford, & Mareschal, 2010), although there is at least one exception (Negen et al., 2019). Unfortunately, with either adults or children, it is not generally clear what separates the cases where cue combination is found from the cases where it is not.

Here, we address the potential role of one important factor that could lead to poor cue combination, in either childhood or adulthood: *internal relative bias*, the difference in a participant's judgments about one world state via two different cues. An internal relative bias is a type of systematic error that results when different sensory processing channels (e.g., a visual and an auditory estimator of object location) are miscalibrated in a way that leaves them unaligned. For example, stimuli that are straight ahead might be perceived as 2° right on average through hearing versus 1° left through vision. The classic normative Bayesian decision-making model (Maloney & Mamassian, 2009; Rohde, Van Dam, & Ernst, 2016) includes a number of assumptions, including that cues are unbiased (both internally and externally). As discussed below, internally biased sensory estimates are very possible, but the status or role of such biases in cue combination has rarely been studied (though see Scarfe & Hibbard, 2011). The central aim of the present studies was to test the hypothesis that higher internal bias is associated with less effective (or even absent) cue combination behavior.

### Key background information

We know that biases can affect cue combination behavior. Not only normatively (reliability-weighted combination is no longer necessarily the optimal strategy given biased estimators) but also experimentally many studies have shown that artificially inducing an *external* bias between two cues can disrupt cue combination behavior (Körding, Beierholm, Ma, Quartz, Tenenbaum, & Shams, 2007; Roach, Heron, & McGraw, 2006; Zhao & Warren, 2015). By external bias, we mean that two cues are physically displaced in the external world (e.g., deliberately by the experimenter) so that they would not indicate the same state of the world to an ideal observer who perfectly recovered their locations. For example, one experiment (Körding et al., 2007) showed a light and played a sound from locations that were offset by 0°, 5°, 10°, or 15°. Participants were asked to judge the position of the sound. As the distance between the two stimuli increased, the position of the light had progressively less influence on their judgments, suggesting a progressive reduction in the averaging of the two signals. This indicates that, when externally biasing one cue away from the other to a sufficient degree, participants will no longer combine them. This effect has been explained in terms of causal inference theory (Shams & Beierholm, 2010): With a large external bias, perception infers that the two stimuli are likely to have different causes and thus it is not informative about the world to combine them. In principle, these principles should equally extend to internal biases, as well, but so far naturally occurring internal biases have been much less studied in the context of cue combination (though see Scarfe & Hibbard, 2011).

### Significance

If internal biases do disrupt cue combination, this could contribute to elucidating two currently unresolved issues in multisensory perception. First, the hypothesis that internal relative bias disrupts cue combination behavior could explain unexpected developmental patterns. Children under 10 years old frequently fail to combine any two cues (Adams, 2016; Burr & Gori, 2011; Dekker et al., 2015; Gori et al., 2008; Jovanovic & Drewing, 2014; Nardini et al., 2008; Nardini et al., 2010; Nardini et al., 2013; Petrini et al., 2014) despite a wide variety of other skills with implicit statistical inference (Gopnik, Meltzoff, & Kuhl, 1999). The proposal that internal relative bias may be higher in early childhood is credible: Sensory systems are still developing and calibrating themselves, all while dealing with major changes to the shape and size of the sensory organs themselves, as well as the dimensions of the body. The central hypothesis might also explain another finding. In one study, unusually, children under 10 years old did combine multisensory cues to location in a setting providing extensive and accurate feedback on single-cue trials (Negen et al., 2019). This feedback could potentially be used to reduce internal relative bias and thus allow cue combination. In summary, internal relative bias might be a key reason why cue combination is so rarely seen in middle or late childhood.

Second, the hypothesis that internal relative bias disrupts cue combination behavior would also shed light on some unexpected and inconsistent findings in adult decision-making. For example, one of the most famous instances of optimal cue combination is the use of a spatialized audio signal and a visual signal to judge horizontal location (Alais & Burr, 2004). That cornerstone study was done with a relatively small group of observers (*N* = 6), and they were not all naïve to the task. Several other studies have failed to replicate a cue combination effect in a variety of similar tasks (Arnold, Petrie, Murray, & Johnston, 2019; Battaglia, Jacobs, & Aslin, 2003; Burr, Banks, & Morrone, 2009; Garcia, Jones, Reeve, Michaelides, Rubin, & Nardini, 2017; Maiworm & Röder, 2011). Biases in auditory localization vary across participants and settings (Dobreva, O'Neill, & Paige, 2012; Odegaard, Wozny, & Shams, 2015) and could have varied substantially in these studies. It is possible that the difference lies in internal relative bias, which may have been lower in that smaller cornerstone dataset. If internal relative bias does disrupt cue combination that could explain why adult audiovisual cue combination is so inconsistent.

The approach here also has important practical applications—for example, to people recovering from sensory loss or brain damage (e.g., stroke) that can lead to a miscalibration of sensory signals and to development of sensory augmentation devices. These efforts will be most effective if supported by an underpinning understanding of the role of calibration of the new signal to those from the existing senses. In addition, such efforts are served by an increasing understanding of brain changes linked to the use of sensory augmentation devices (Amedi, Hofstetter, Maidenbaum, & Heimler, 2017). The information gathered here may also create insights into the principles that govern some of those changes.

### Specific research gaps

The primary research gap addressed here is an empirical test of how internal biases relate to cue combination behavior. The key question is whether relative bias can disrupt cue combination. There is already theory that explores a link between internal bias and disrupted cue combination (Burr & Gori, 2011), at least in developmental contexts, but it is not yet supported by measurements of internal biases. Several studies with adults have measured relative bias and/or multisensory performance but have not gone as far as explicitly testing for a link between internal relative bias and cue combination (Odegaard et al., 2015; Scarfe & Hibbard, 2011; Smeets, Van Den Dobbelsteen, De Grave, Van Beers, & Brenner, 2006). This leaves a significant empirical gap.

The secondary research gap addressed here is simply a measurement of internal biases in a developmental sample. A key question is whether children do indeed have large biases. When adults carry out audiovisual localization, it is well established that some patterns of biases are likely to present themselves (Dobreva et al., 2012; Odegaard et al., 2015). Because of the very limited measurement of biases hitherto in developmental studies, it is much less clear whether we should expect anything similar in middle or late childhood on any of the tasks that have so far failed to show cue combination. In other words, even if we grant that internal relative biases do disrupt cue combination, it is not yet clear that children do have the large biases that, together with this, could contribute to explaining changes in cue combination behavior during middle and late childhood.

### Main hypothesis

We hypothesized that high internal relative bias is associated with less-effective cue combination. For the avoidance of any doubt, *internal relative bias* is the difference in a participant's judgments about one world state via two different cues. For example, a participant who, on average, reports physically co-located auditory versus visual stimuli as being at 10° and 15°, respectively, has a relative bias of 5°. For the purposes of testing whether magnitude of bias is associated with cue combination, we express internal relative bias as an absolute value (does not have a direction and cannot be negative). We focus here specifically on *relative* bias (perception via cue 1 vs. perception via cue 2) rather than *absolute* bias (perception vs. veridical). In the above example, the real physical stimuli were co-located, but they might equally each have been at 10°, 15°, in-between, or elsewhere entirely. Within a task presenting only those two cues, the observer has no way to verify or correct this, as they have access only to their own slightly conflicting percept of 10° (auditory) and 15° (visual). Paralleling the approach in the causal inference literature (Körding et al., 2007), in which relative biases were induced externally, we take this subjectively experienced relative bias as the key input to cue combination behavior about which predictions can be made.

### The present study

Both experiments were designed to examine the relation between cue combination and relative bias. Our key prediction is that, when we measure internal biases, lower internal biases will be associated with greater cue combination precision gains and vice versa. To determine this, we extend the classic cue combination approach: In addition to the standard tests for use of different single and combined cues, we also measure the extent to which participants’ judgments of one world state via two different single cues are biased relative to each other.

In Experiment 1, we considered the case of children's three-dimensional (3D) slant perception via disparity and texture, a system previously shown to be immature through childhood (Nardini et al., 2010), although near-optimal from 12 years and in adulthood (Hillis, Watt, Landy, & Banks, 2004; Nardini et al., 2010). Here, we took an individual-differences approach by examining cross-sectional correlation between internal relative bias and cue combination behavior. In Experiment 2, we considered the case of adults’ visual-auditory localization in azimuth, a system with inconsistent previous cue combination findings (Alais & Burr, 2004; Arnold et al., 2019; Battaglia et al., 2003; Burr et al., 2009; Garcia et al., 2017; Maiworm & Röder, 2011). Here, we took a training approach: We tested to see if an intervention designed to reduce internal relative bias would promote cue combination behavior. Two different tasks and populations were used in order to test for a link between cue combination and internal relative bias as a general principle, not one that might be present in just one specific setting. The populations tested in each experiment (Experiment 1, children; Experiment 2, adults) were those previously showing inconsistent cue combination in that setting, as reviewed above.

## Experiment 1

**Figure 1.**
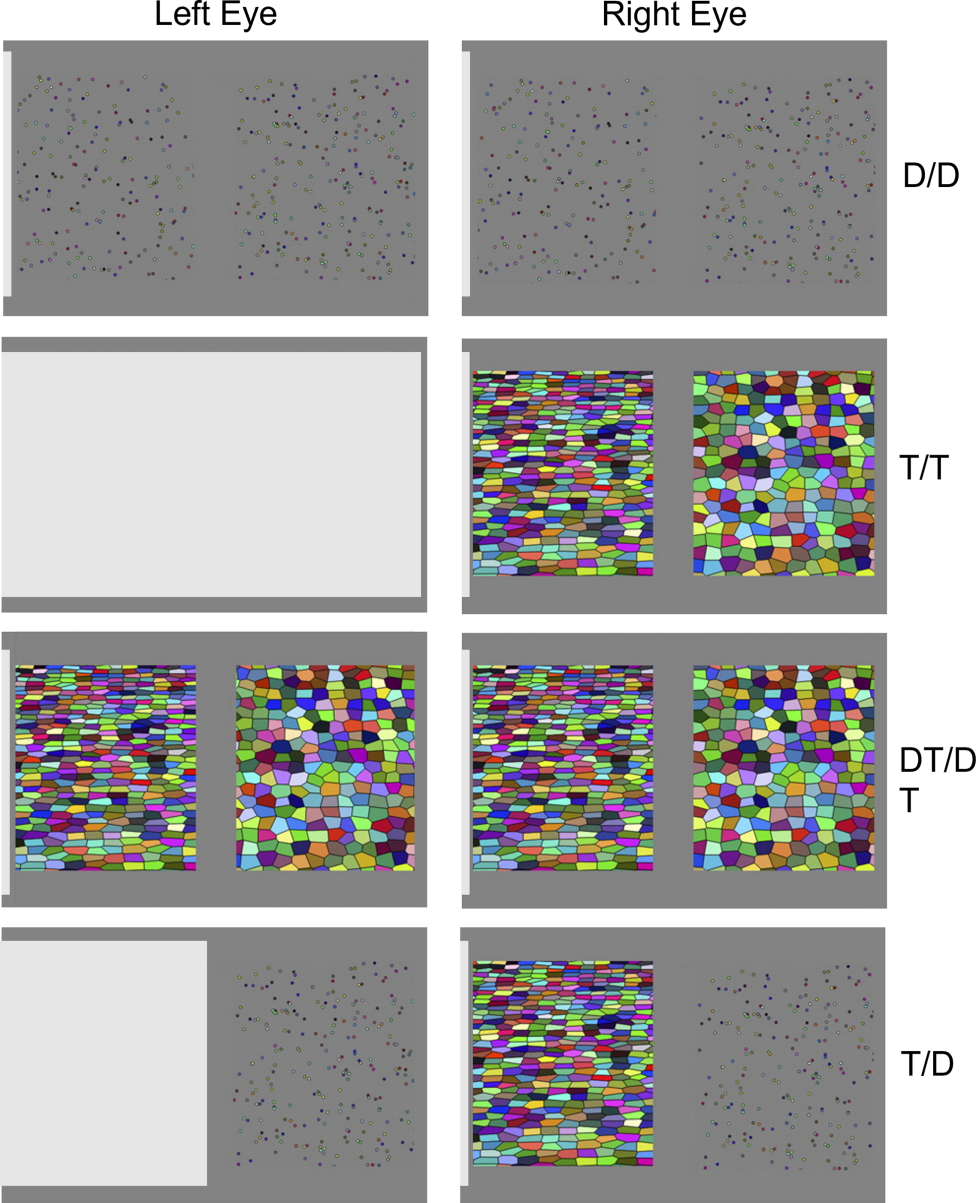
Example stimuli. Shutter glasses were used to show different views to the left and right eyes (left and right columns). D represents disparity cue, and T represents texture cue. The top three rows test for cue combination and the bottom tests for internal relative bias.

Experiment 1 tested the prediction that children ages 7 to 10 years, judging 3D surface slant via disparity and/or texture (see Figure 1 for examples), would show a negative cross-sectional correlation between internal relative bias and cue combination precision advantage. That is, children whose disparity-based versus texture-based judgments of slant are less biased relative to each other would make greater precision gains via combination of those two cues to slant. We considered that this effect may be separable from an age effect, depending on the strength of the collinearity between age and relative bias. We further considered that any internal relative bias shown by the participants could be used as a “screen” to identify a subset of children who were able to combine cues in a task where the larger sample does not do so on average. To prevent learning of either relative bias or the correct cue combination method, and as is standard in most cue combination studies, no feedback was provided.

We chose this population and task because we expected good variation in both internal relative bias and cue combination behavior—features that would help with measuring a correlation between them. Internal relative bias should vary here because the eyes, skull, and surrounding tissues of these participants are still growing and often doing so quickly (Mann, 1984). Such physical changes could disrupt the calibration of visual slant cues and create bias if the effects are unequal on different cues (e.g., a sudden change in the distance between pupils might have a particular effect on disparity). These considerations suggest that there could be biases during depth perception via different cues in childhood, although the existence or magnitude of such biases has not been measured before—in itself an important innovation of this study. In addition, previous research suggests that cue combination behavior is likely to be highly variable. Previous studies with the same task and stimuli did not find significant cue combination precision advantages at 6 to 10 years, only from 12 years and in adults (Hillis et al., 2004; Nardini et al., 2010). A related study of two depth cues (motion and disparity cues to depth) with a continuous age range found a gradual emergence of average cue combination behavior around the 11th year of life, but substantial individual variation in this over the whole 6- to 12-year age range (see figure 2 in Dekker et al., 2015). Taken together, the plausibility of biases and the likelihood of substantial individual variation in cue combination behavior makes this system a strong candidate for testing for a cross-sectional correlation between internal bias and cue combination behavior.

**Figure 2.**
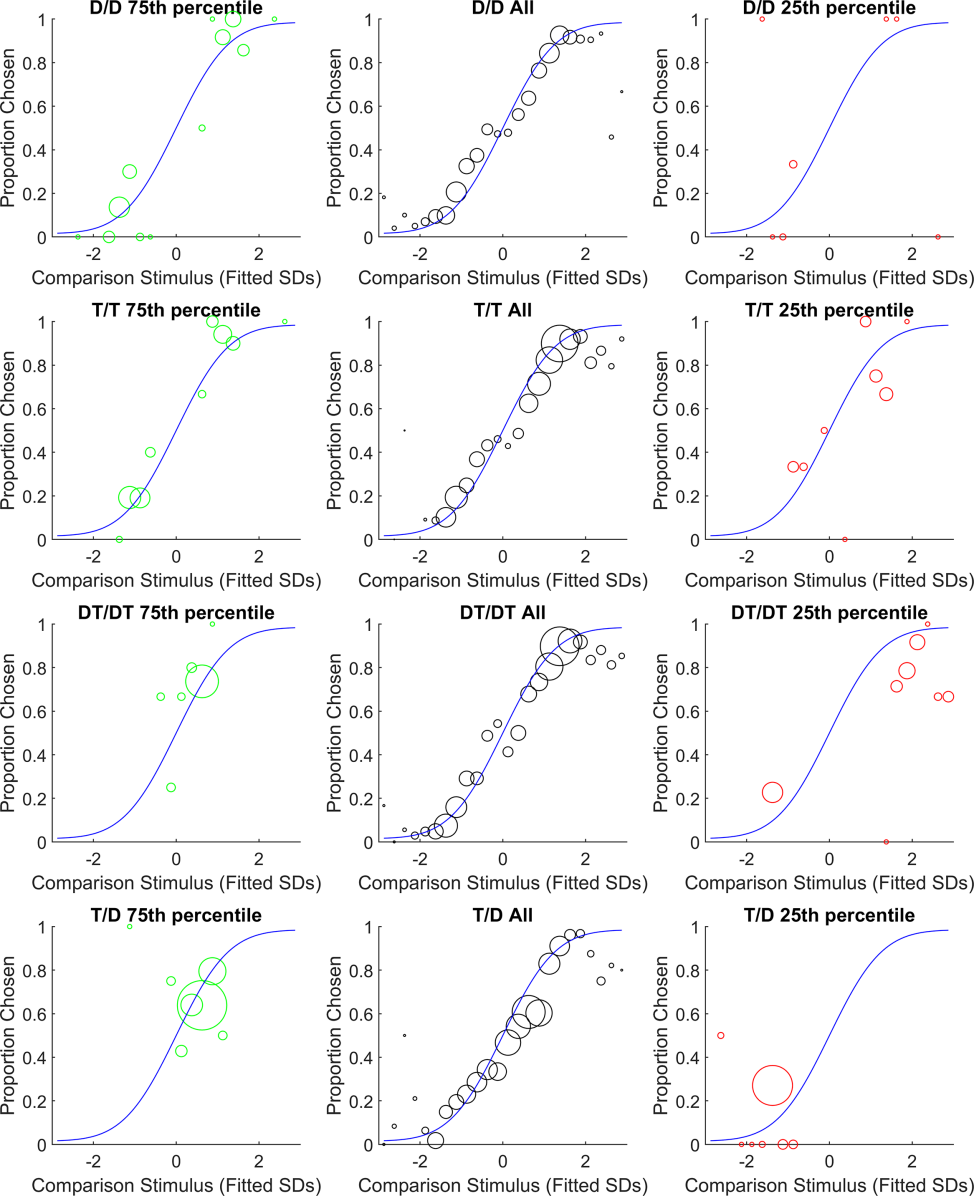
Goodness of fit for the model in Experiment 1. The circles are observations, and the blue lines are model fits. The *x*-axis is the difference between the alternative stimulus and the standard (45°) expressed in units of the fitted standard deviation for the choice. The *y*-axis is the proportion of trials where the alternative was chosen. The size of each black circle is proportional to the number of observations in its range. Each circle was calculated with a range 0.25 *SD* wide. The center column pools all participants together. The left column shows a participant with a relatively good fit and the right column shows a relatively poor fit.

### Method

The core task was to look at two stimuli, one surface on the right side of a screen and one on the left, and to indicate which appears more deeply slanted (i.e., closer to a horizontal table top) (Figure 1).

#### Participants

Participants were a sample of 95 children (48 males) 7 to 10 years of age. They were recruited in local schools around Durham in the United Kingdom. An additional seven participants were excluded because they either could not perceive the stereo depth or did not complete all of the trials. The final sample had nine children 7 years old, 30 children 8 years old, 32 children 9 years old, and 24 children 10 years old. Written informed consent was obtained from parents, and verbal assent was obtained from the participants themselves. The study was approved by the Ethics Committee in the Psychology Department at Durham University (reference 16/07–Durham Multisensory Learning Project). Interpupillary distance was measured for each individual and used to calculate correctly projected stereoscopic stimuli.

With 95 participants, power for the critical correlation is expected to be 80% for a standardized beta of at least 0.25, 95% for a slope 0.32, and 99% for 0.38. Although the expected slope could not be meaningfully estimated, we felt that explaining less than 5% of the variance (standardized beta of 0.25) was a reasonable minimum level of theoretical interest. In addition, a previous project suggests that near-optimal cue combination effects in this age range tend to be relatively large, over *d* = 1.0 (Negen et al., 2019). This requires only 13 participants for 95% power. We were planning to do a median split and look for differences between the two groups, with one expected to have a reasonably strong cue combination effect and another expected to have an absent/minimal cue combination effect. We estimated this as a between-group difference of *d* = 0.6, which provided 90% power with 95 participants.

#### Apparatus

Stimuli were presented on a liquid-crystal display (LCD) screen (Model 2233RZ; Samsung, Suwon, South Korea). Testing was conducted on a GeForce 3D Vision system (NVIDIA Corporation, Santa Clara, CA), which included a pair of active shutter glasses (P854) with separate control over the shutter on each eye and a small wireless infrared emitter that sends a synchronization signal from the computer to the glasses (P701). Synchronizing the shutters and the 120-Hz display, the system displays alternate frames to each eye, allowing for stereoscopic stimuli. Beyond this, only a standard keyboard was used, and the left and right arrow keys were used to respond.

#### Stimuli

Stimuli were based closely on a previous experiment (Nardini et al., 2010). Each stimulus was composed of two planes with a certain slant. Each plane was projected as if seen through a 18.5 × 25-cm aperture in a gray screen at the same distance as the monitor screen; thus, the planes were projected as if they were behind the monitor. The edges of the planes could not be seen. Observers’ eyes and the horizontal axis about which planes rotated were aligned with the vertical center of the monitor. The distances in depth of the planes were each 15 to 35 cm from the screen; in other words, the planes appeared as though deeper inside the screen. Disparity and/or texture cues signaled slants about the horizontal axis, away from the observer. The binocular cue to slant came from the gradient of interocular disparities. Because slants were about the horizontal axis, horizontal and vertical size ratios were not useful, and, as stimuli were projected on a flat screen at a fixed distance, vergence and accommodation did not usefully covary with slant. Because the previous project had already roughly matched these two stimulus types in precision (Nardini et al., 2010), there was no need for further piloting to match them.

##### Trial types

Three trial types were designed to help measure cue combination. The first included trials in which both the left and right side had a disparity cue (D/D trials). The second type included trials in which both the left and right side had a texture cue (T/T trials). The third trial type included both the left and right disparity and texture cue (DT/DT trials). The final trial type was designed to measure relative bias (T/D trials). In these trials, the left side had a texture cue and the right side had a disparity cue. This choice was necessary so that the monocular information was always fed to the right eye, which dampens any additional variability in responses that could arise from participants with different levels of monocular acuity in the two eyes.

Using a finite, preselected subset of all the possible stimuli is important for Quest+ to work properly. For every trial, one side (the standard) cued a slant of 45°. The other side (the comparison) could vary from 20° to 70° in increments of 1°. This results in a table of 408 possible stimuli (4 types × 51 of the non-45 comparison slants × 2 sides that the non-45 comparison slant could be on).

##### Projection of the planes

Planes were first composed of a regular grid of points with average density of 0.6 points/cm (±0%–10% for each plane on each trial) in both *x* and *y* directions. When a plane only had a disparity cue, the points were rendered as randomly colored dots 0.2 cm wide. They had appropriate disparities signaling slant but not the changes in dot size and density that would provide a texture cue to slant. In other words, there was no change in average size or density around the two-dimensional (2D) space of the screen. To further reduce the appearance of a grid or texture in these stimuli, the points were randomly jittered 1.5 grid squares (2.5 cm), which effectively produces a random spread (Figure 1). Although it remains theoretically possible that this would still be interpreted as a weak cue to a vertical plane—the texture and size do not change in density over the vertical axis—such a cue would be quite weak with such extreme spread. We expect this cue, if perceived, would have negligible effects on the outcomes here.

When a plane had a texture cue (i.e., T or DT), each point formed the center of a randomly colored tile with vertices defined by a Voronoi tessellation. In these cases, the jitter was reduced to ±0 to 0.3 grid squares (0.5 cm), resulting in a random but orderly texture (Figure 1) in which slant can be judged by changes in this texture. As slant increases, the tessellated elements become denser, and their outlines converge in line with perspective.

For the monocular texture cue, we used a virtual version of an occluder on the left eye. The left eye did not receive an image of the plane itself but rather a gray square with a straight edge. The right eye also had the far edge of the gray square in its far left margin so as to match a plausible object near the face. This produced an effect similar to a gray piece of paper near the observer fully or partially obscuring the left eye, meaning the participant could view the whole screen with the right eye but only part of the screen with the left eye. For T/T trials, the gray square fully obscured the display, so that neither stimulus was visible to the left eye. For T/D trials, the gray square was positioned so that the left eye could only see the right half of the screen (the part with the disparity cue). For D/D trials and DT/DT trials, the gray square was only partially visible at the left edge of the display, so that both eyes could see both stimuli. In addition to the introduction of the novel T/D trial type, the use of the virtual occluder is the second way that our stimuli diverge from the previous project (Nardini et al., 2010). In the previous project, it was never necessary to block only part of the screen. We required a virtual occluder here to precisely present the T/D trials and introduced it into other conditions for consistency across the trial types. When a plane had both cues (i.e., DT trials), the Voronoi tessellation was projected stereoscopically to both eye, which provided both the perspective information in the texture cue and the stereo information in the disparities.

#### Procedure

Participants were seated 175 cm from the screen and were presented with a total of 300 trials. The first six trials were always the same. The first two were D/D trials, comparing 45° to 20° and then 45° to 70°. Participants were asked if the dots looked like they went backwards into the screen, slanted down. Those who could not see this were excluded. The next two were T/T trials, making the same comparisons. The last two were DT/DT trials, again making the same comparisons. These initial easy trials were used to familiarize participants with the task. After this, Quest+ was allowed to select the next trial. Every trial had one side that was slanted by 45°.

On every trial, the participant was asked which of the two stimuli, left or right (Figure 1), was more slanted. This was explained to participants by pointing to a door and saying it is all the way “up” and then pointing to the surface of a table and saying that it is all the way “down.” The goal is to say which is more “down,” like the table. Hand gestures were used to emphasize the slant aspect.

##### Quest+ for stimulus selection

Stimuli were determined with a tailored Quest+, which is a general procedure for determining the next stimulus based on prior assumptions and previous responses (Watson, 2017). In a Quest+ design, there is a model with specific parameters. The parameters have a predetermined set of different settings that they can take, called a quantization. The goal of the procedure was to concentrate as much posterior probability as possible onto one setting for the parameters. The stimuli were chosen adaptively during the experiment based on which parameters were most likely to accomplish this goal. (When two trials have equal estimated utility, Quest+ chooses randomly.) This allowed for a large gain in the efficiency of estimation, meaning good parameter estimates could be obtained with far fewer trials. As Quest+ can continue picking new trials indefinitely, it was set to terminate after 300 trials.

##### Quest+ model parameters

The Quest+ model had four parameters. The first was σ_D_, the standard deviation (*SD*) of estimates with a disparity cue. The second was σ_T_, the standard deviation of estimates with a texture cue. The third was σ_DT_, the standard deviation of estimates with both cues. These were all quantized from 2° to 30° on a logarithmic scale with 16 steps. Evidence for cue combination can be found when σ_DT_ is smaller than σ_D_ and smaller than σ_T_. The fourth parameter was *B*, the relative bias. This was the difference in the average degrees of slant perceived when both disparity and texture signaled the same slant. A positive value of *B* meant that the disparity cue was perceived as slanted more (“down”) than an equal texture cue. This was quantized from −40° to +40° with 16 steps. This resulted in a table of 65,536 quantized parameter settings (16^4^).

##### Quest+ model mechanics

Comparing the left (L) side and the right (R) side with mean perceived slant of µ_L_ and µ_R_, respectively, given standard deviations in their perceptions as σ_L_ and σ_R_, we modeled the probability of choosing side L as

(1) $$\begin{array}{c} \Phi \left(\frac{\mu_{L}-\mu_{R}}{\sqrt{\sigma_{L}^{2}+\sigma_{R}^{2}}}\right)\times \;0.97+0.015\; \end{array}$$

where Φ is the standard normal cumulative distribution function (CDF). The 0.97 factor and 0.015 constant act as a lapse mechanism; the modeled participant always has at least a 1.5% chance of choosing a given side. This dampens the effect of outlier trials. In the case of a D/D, T/T, or DT/DT trial, µ is simply the correct slant on the relevant side. In the case of a T/D trial, the disparity side has *B* added to the correct slant to calculate µ_R_. For a D/D trial, σ is equivalent to σ_D_. The same is true for the disparity side of a T/D trial. However, for trials involving texture, there is an issue to address with the consistency of perceptual noise. As slant increases (toward the horizontal), the texture cue becomes increasingly reliable because a set amount of rotation of the plane about its axis makes more and more difference to its perspective projection (Blake, Bülthoff, & Sheinberg, 1993; Knill, 1998). When a trial includes texture information, we therefore adjust the σ used as

(2) $$\begin{array}{c} \;\sigma e^{-0.03\left(\mu -45\right)}\;\; \end{array}$$

where µ is the correct slant and σ is the unadjusted standard deviation. The 45 constant here is simply to center it on the midpoint of the stimulus range (45°). This would adjust a standard deviation of 1° to a standard deviation of 0.472° at a slant of 70°. It would also adjust it to a standard deviation of 2.11° at a slant of 20°. The multiplier −0.03 for the exponent provided a good fit to data in an earlier study (Nardini et al., 2010).

To be specific, the probability of choosing side L on a D/D trial is

(3) $$\begin{array}{c} \Phi \left(\frac{L-R}{\sqrt{\sigma_{D}^{2}+\sigma_{D}^{2}}}\right)\times \;0.97+0.015\; \end{array}$$

where *L* and *R* are the slants of the left and right planes, respectively.

The probability of choosing side L on a T/T trial is

(4) $$\begin{array}{ccc} & & \Phi \left(\frac{L-R}{\sqrt{{\left(\sigma_{T}e^{-0.03\left(L-45\right)}\right)}^{2}+{\left(\sigma_{T}e^{-0.03\left(R-45\right)}\right)}^{2}}}\right)\\ & & \;\times \;0.97+0.015\; \end{array}$$

The probability of choosing side L on a DT/DT trial is

(5) $$\begin{array}{ccc} & & \Phi \left(\frac{L-R}{\sqrt{{\left(\sigma_{DT}e^{-0.03\left(L-45\right)}\right)}^{2}+{\left(\sigma_{DT}e^{-0.03\left(R-45\right)}\right)}^{2}}}\right)\\ & & \;\times \;0.97+0.015\; \end{array}$$

The probability of choosing side L on a T/D trial is

(6) $$\begin{array}{c} \;\;\;\;\;\;\;\;\;\;\;\;\;\Phi \left(\frac{L-R-B}{\sqrt{{\left(\sigma_{T}e^{-0.03\left(L-45\right)}\right)}^{2}+\sigma_{D}^{2}}}\right)\times \;0.97+0.015\; \end{array}$$

The prior distribution on each σ was gamma (2, 15). This was chosen because gamma distributions are strictly non-negative, these specific distributions are centered near the previous project's average measurements, and they have a wide variance. The prior on *B* was normal (0,10). This was chosen because the bias requires support over the whole real line (with positive and negative values indicating bias in opposite directions), it is centered in a way that supposes either bias direction a priori equally, and it has a wide variance. As it happens, these priors mostly serve to help initialize the Quest+ procedure and are quickly outweighed by the actual data.

##### Quest+ customizations

We also disallowed some extreme levels of sampling specific trial parameters. For DT/DT trials, we did not allow more than 2/3 of trials to have a comparison side with a slant of more than 45°. We also did not allow more than 2/3 of trials to have a comparison side with a slant of less than 45°. The same rule was applied to T/T trials. This was done to be sure that the correct answers were roughly evenly distributed across the comparisons versus standards. We also did not allow the number of any one trial type (D/D, T/T, DT/DT, or T/D) to fall below 15% of the number of trials. This was done to be sure that at least some amount of relevant data were gathered for every trial type.

##### Calculated variables for analysis

After the data were collected, the four parameters in the Quest+ algorithm were fitted for each participant via a maximum likelihood method. This was done with the *fminsearch* function in MATLAB (MathWorks, Natick, MA); that is, there was no analytic formula that governed the estimate. The algorithm finds the values of *B*, σ_D_, σ_T_, and σ_DT_ that lead to the highest joint probability of each participant's data. Note that this produces estimates that are no longer constrained by the quantization (see Quest+ model parameters). The internal relative bias was then calculated as the absolute value of *B*; in other words,

(7) $$\begin{array}{c} \mathrm{Internal}\;\mathrm{relative}\;\mathrm{bias}=|B|\; \end{array}$$

The cue combination effect, a bimodal precision advantage, was calculated by taking the precision (1/variance) with both cues and subtracting the precision with the best single cue for each participant.

##### Goodness of fit

A basic examination of the model fit did not find any obvious or large problems. Figure 2 charts observed comparison choice proportions against fitted comparison choice proportions using bins of 0.25 *SD*. The center column pools all participants, and the left column and right columns show individual participants. These individual participants are the 75th percentile (relatively good; left) and 25th percentile (relatively bad; right) fits as measured by mean absolute difference between predicted and observed counts. Overall, the predicted count of comparison choices in each bin explained over 99% of the variance in observed count of comparison choices in each bin. There are a few places where the observed proportion deviates from the fitted proportion by more than 10%, which could be alarming. However, this appears only to have happened in instances where the number of observations was relatively small. It does not appear to have disrupted the ability of the model to reasonably capture the major trends in the data—perhaps due to the lapse mechanism.

### Results

**Figure 3.**
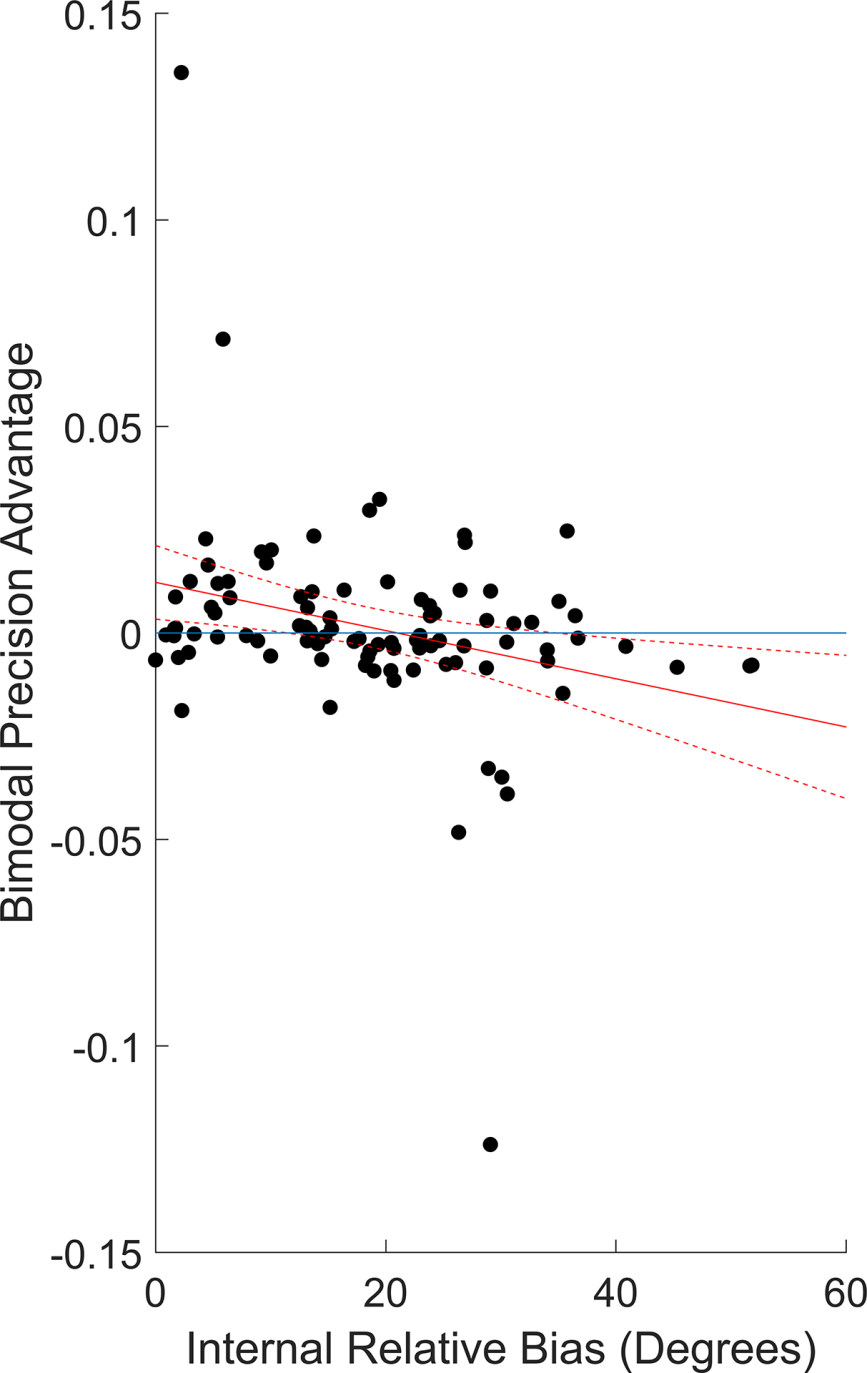
Relation between fitted measures. Black dots are individual participants, the solid red line is a line of best fit, and the dashed red lines are a 95% CI around the line of best fit. The blue line is a reference line at zero. Conforming to the predictions, internal relative bias was a significant negative predictor of bimodal precision advantage (a measure of cue combination behavior). This was also true when ranks were analyzed rather than raw values.

Data are attached as Supplementary Material (for both experiments). Results conform to the prediction: Greater internal relative biases were associated with less effective cue combination (Figure 3). In a multiple linear regression with bimodal precision advantage as the outcome, age was not a significant predictor, *t*(92) = −0.06, *p* = 0.953, standardized beta = −0.006 (95% confidence interval [CI], −0.204 to 0.193), but relative bias was a significant negative predictor, *t*(92) = −2.87, *p* = 0.005, standardized beta = −0.287 (95% CI, −0.485 to −0.088). Overall *R*^2^ = 0.0824 for both predictors. Examination of the bimodal precision advantage outcomes suggests that this may have an issue with outliers (*z*-scores of −5.2, 2.9, and 5.6; all others less than 2.5), so we will also report non-parametric results. When entering ranks instead of raw values, the age effect was not significant, *t*(92) = 0.72, *p* = 0.476, standardized beta = 0.071 (95% CI, −0.126 to 0.268), and the bias effect was significant, *t*(92) = −3.10, *p* = 0.003, standardized beta = −0.308 (95% CI, −0.504 to −0.111). Overall *R*^2^ = 0.0984 for both predictors. This suggests that lower internal relative bias is cross-sectionally correlated with cue combination behavior and that this is not particularly explained by age differences.

**Figure 4.**
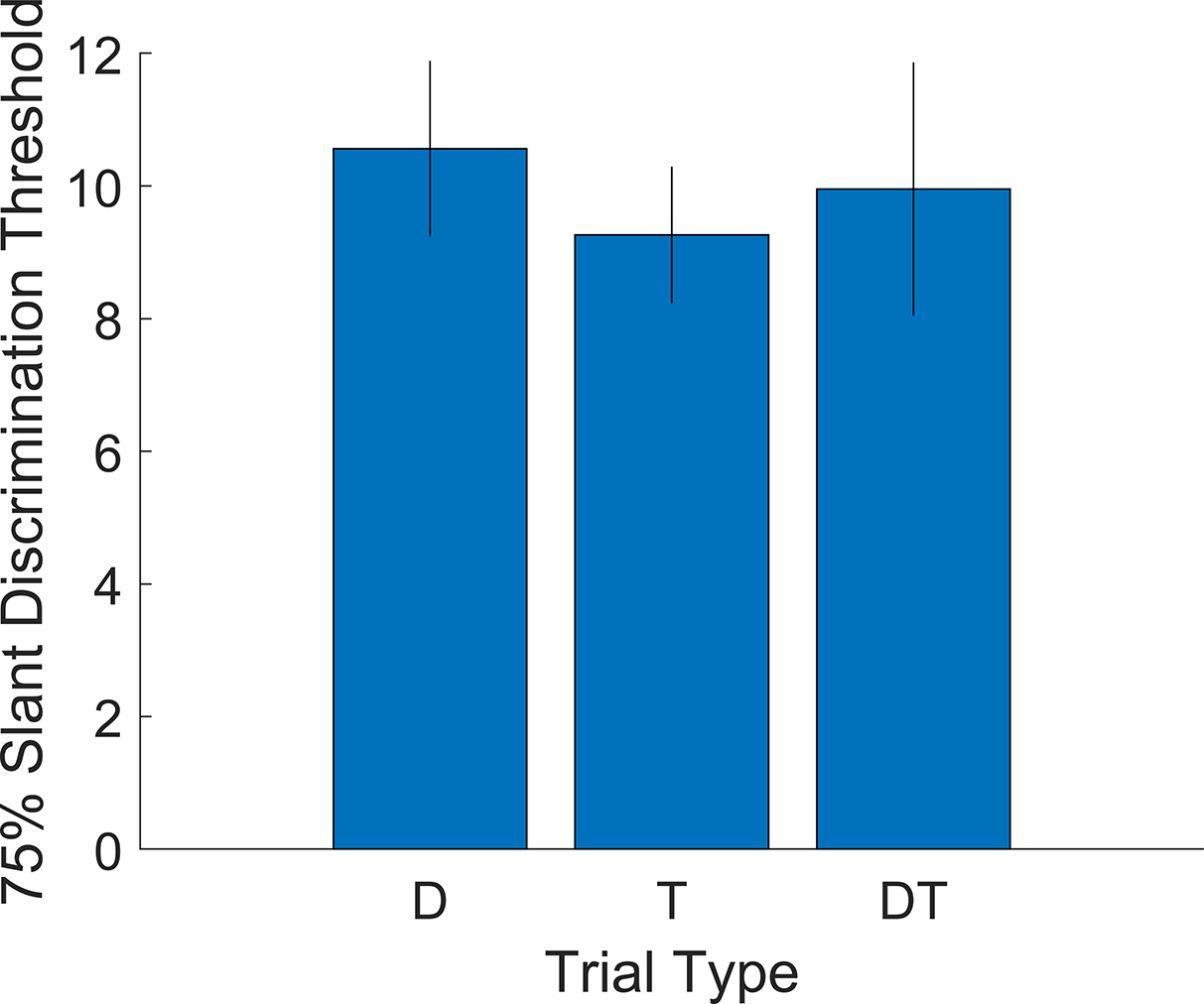
Slant discrimination thresholds in degrees by trial type, calculated and displayed in the same way as a similar previous project for comparison.

We also checked the more basic descriptive results against the previous project with similar methods (Nardini et al., 2010). The fitted thresholds were also broadly similar (Figure 4), though slightly higher here. An analysis of overall cue combination also agrees with the previous project. Over the entire sample, precision with both cues was not significantly different from precision with the best single cue, *t*(94) = 0.54, *p* = 0.588, signed-rank test *p* = 0.601. These results are consistent with many previous reports that failed to detect a significant bimodal precision advantage in children at 7 to 10 years old using stimuli and a task like those here (Nardini et al., 2010) and in other, multisensory settings (Adams, 2016; Burr & Gori, 2011; Dekker et al., 2015; Gori et al., 2008; Jovanovic & Drewing, 2014; Nardini et al., 2008; Nardini et al., 2013; Petrini et al., 2014).

**Figure 5.**
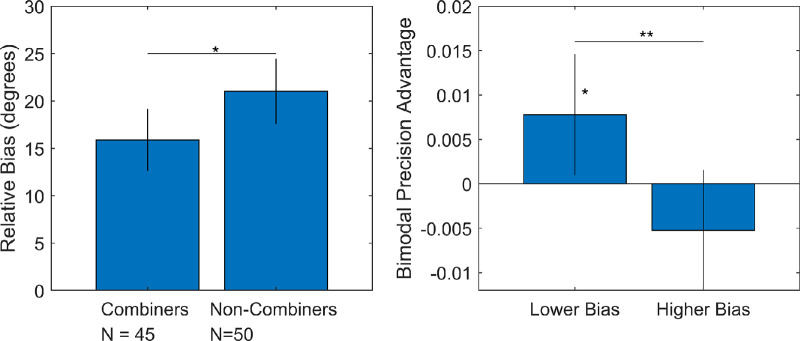
Relative bias and cue combination in Experiment 1. On the left, participants were sorted into combiners (single-cue precision less than dual-cue precision) and non-combiners (single-cue precision equal or greater than dual-cue precision). Relative bias was significantly higher in the non-combiners. On the right, those with below-median bias had a significantly above-zero bimodal precision advantage (i.e., combined cues), whereas those with below-median bias did not. In addition to correlations in the text, these results suggest that internal relative bias had a negative cross-sectional relation with cue combination behavior.

In addition, we also carried out a more categorical version of the analysis. The step difference between zero bimodal precision advantage and positive bimodal precision advantage is theoretically meaningful, indicating a difference in algorithm. We classified participants as “combiners” if their estimated precision with both cues was higher than either single cue and as “non-combiners” otherwise (45 combiners vs. 50 non-combiners; mean ages, 9.26 vs. 9.23 years). Combiners had a lower relative bias than non-combiners, *t*(93) = −2.14, *p* = 0.035, rank-sum test *p* = 0.028 (Figure 5). Note that even in the combiners group, mean relative bias was still well above zero.

To continue the categorical analysis, participants were subject to a median split by relative bias amount (18.6°). Among those with below-median relative bias, precision with both cues was higher than with the best single cue for each participant, *t*(47) = 2.29, *p* = 0.026, signed-rank test *p* = 0.022. This suggests that 7- to 10-year-olds with a lower level of relative bias do combine these two cues to improve their precision at judging slant. Among those with above-median relative bias, precision with both cues was not significantly different from precision with the best single cue, *t*(46) = −1.55, *p* = 0.129, signed-rank test *p* = 0.130. Further, the difference in bimodal precision advantage (precision with both cues minus precision with the best single cue) was significantly different in participants with below-median relative bias versus above-median relative bias, *t*(93) = 2.72, *p* = 0.008, rank-sum test *p* = 0.003. This is an unusual result in the developmental literature, as we found successful precision improvements consistent with cue combination, specifically in those 7 to 10 years old with lower relative bias. This improvement was not seen in those with higher bias, and the measure differed significantly across groups.

In addition to these planned analyses, we also checked for a relation between cue combination behavior and interpupillary distance. This was not significant, *r*(93) = 0.13, *p* = 0.218. In other words, we did not find evidence for a direct relation between physical size and cue combination. Future study may be more able to directly address this sort of issue with more extensive (ideally longitudinal) physical growth outcomes.

### Discussion

The data from Experiment 1 are consistent with the prediction. A link between internal relative bias and cue combination was found in several ways: The measures were correlated, even when controlling for age, and the children with below-median bias had a significant cue combination effect, whereas the children with above-median bias did not, with a significant difference between them. In addition, the children who combined cues had lower relative bias than the children who did not. This could suggest that a high level of internal relative bias is a key factor to consider when accounting for why children under 10 years old fail to combine cues.

This further suggests that internal relative bias has at least some measurable and interpretable level of variation in childhood. The magnitude of the internal relative bias that was measured here might be surprising; in a small number of participants, it reached over 45°. We should caution here that, although the procedure tried to efficiently estimate bias and imprecision parameters, the number of trials per participant was not very high by psychometric standards. This means that some random measurement error is almost certainly present. In other words, it cannot really be known if the highest estimates reflect actual biases of that size. However, such measurement error would only bias the experiment away from the conclusions reported here. Because the results still show a correlation, even in a non-parametric version of the analysis, we feel confident that there is some meaningful variation in internal relative bias in this population and this task.

For a reader who is more familiar with the adult literature on cue combination, the presence of negative bimodal precision “advantages” may require explanation. These advantages are well known and have been documented in studies of children under 10 years of age. Such negative advantages at a group level are evident, for example, in young children's visual–haptic size judgments (Gori et al., 2008) and visual–vestibular navigation accuracy (Nardini et al., 2008). A negative advantage occurs when the presentation of a second cue fails to improve performance but instead harms performance. This is consistent with over-weighting or over-relying on less reliable cues. There is nothing about these tasks that forces young participants to use the more reliable cue if they have two available.

These results are entirely consistent with the main hypothesis but they have the limitations common to all individual differences studies. First, because correlation does not imply causation, the data cannot show that larger internal biases *cause* poorer cue combination behavior. This interpretation is strongly supported by earlier research on cue combination, particularly the effects of inducing biases (Körding et al., 2007; Roach et al., 2006; Zhao & Warren, 2015). However, it remains possible that, in this population, some other common factor or causal structure accounts for some of the link we observed. Second, because the individual differences approach studies variation that occurs naturally, rather than inducing variation through experimental manipulations (e.g., here, random assignment, somehow, to a high-internal-bias or low-internal-bias condition), it remains possible that some other unknown dimensions of variation correlated with the one of interest account for some of the link. We therefore consider this evidence highly consistent with the overall proposal but necessarily limited by the individual differences/correlational approach. This aside, our study of natural individual differences clearly reveals a new and important aspect of cue combination development, that in a group who on average do not combine cues there is a subgroup who do, identifiable by having low relative bias. This new consideration of individual differences leads to a rethink of how many previous studies of cue combination in childhood have been interpreted (Adams, 2016; Burr & Gori, 2011; Dekker et al., 2015; Gori et al., 2008; Jovanovic & Drewing, 2014; Nardini et al., 2008; Nardini et al., 2010; Nardini et al., 2013; Petrini et al., 2014) (see above). To better understand the role of internal biases in cue combination, Experiment 2 examines this question from another angle—a different population, a different task, and a different study design.

#### Coordination note

The two experiments in this paper use slightly different calculation methods to measure the critical cue combination effect. Our core principle in this decision is that we wanted to use preplanned analyses whenever there was not a strong scientific reason to indicate against it. The analyses presented above were all planned, programmed, and archived by the first author before the data were collected. The analyses below were planned, programmed, and preregistered before the data were collected. After all data were collected for both, we realized that the two use calculations that are slightly different. Specifically, Experiment 1 subtracts the precision (1/variance) of the best single cue from the precision with both cues. Experiment 2 subtracts the variable error (VE; standard deviation) with both cues from the VE with the best single cue. With enough data, both are valid ways of testing for cue combination effects; one looks for an increase in precision, and the other looks for a decrease in standard deviation. For future projects, we recommend the method used in Experiment 2 because, in our experience, it is generally closer to a normal distribution. As it happens, the multiple regression reported above has the same pattern of significance when switching to a VE outcome.

## Experiment 2

Experiment 2 tested the prediction that adults, when asked to judge a horizontal location using an audiovisual stimulus, would increase their cue combination behavior after a short training intervention that decreased their internal relative bias. We first asked adults to judge a horizontal location with an audio cue or a visual cue, or both. This was done to measure their internal relative bias and their cue combination behavior. A short training period was then given during which participants received accurate feedback on audio-only trials and visual-only trials. This was designed to calibrate their perception of location via these two separate cues (i.e., to achieve a lower relative internal bias). If internal biases limit cue combination, then a successful reduction in relative bias via our method should be accompanied by an increase in cue combination behavior. We tested this by again asking participants to judge a horizontal location with an audio cue, a visual cue, and both. No feedback was be given for the audiovisual trials, meaning that any improvements in performance on those trials could not be explained by directly training the participants on the best method of completing such trials. Because this procedure is a within-subjects one, it would not be possible to conclude that a positive result was due to unmeasured differences among the subjects.

The experiment was preregistered at https://osf.io/eb7yf/wiki/home/. We predicted specifically that cue combination would be greater in block 3 than block 1 (i.e., post-training vs. pre-training), that it would be significantly above zero in block 3 (i.e., post-training), and that it would be related to relative bias in a linear regression.

### Method

During each trial, participants were required to identify the location of a small hidden cartoon dog along the horizontal (left/right) axis of a large screen.

#### Participants

Forty participants were tested, and 39 were included (one was removed for failure to show a correlation between target and response). When asked to indicate their gender, 10 people selected male, 28 people selected female, and one person selected “non-binary or prefer not to say.” Average age was 21.85 years. The age distribution had a large right tail; 36 participants were between 18 and 23 years old, and the remaining three were 25, 45, and 71 years old. Participants were given either £10 or 1 hour of participant pool credit, a system where psychology students are rewarded for participating in studies by gaining credits to award to participants for their own future studies (not a course credit). This study was approved by Durham Psychology's Ethics Board (Reference: PSYCH-2018-12-04). Informed consent was given by participants in writing.

The experiment used a statistical technique that is well known in the medical literature: the Pocock boundary for two planned analyses (Pocock, 1977). In this procedure, the first step is to decide on and preregister the critical analysis (or analyses). Next, half of the final sample is gathered. This is tested with an alpha value of 0.0294 (Pocock, 1977), assuming that one wants to control the overall type I error rate at 5%. If all *p* values are below 0.0294, testing ends and the null hypothesis (or hypotheses) is rejected. If not, the other half of the sample is gathered (as occurred here). The entire sample is again tested with an alpha value of 0.0294. This has the advantage of both (a) controlling the type I error rate at 5% overall and (b) allowing for the possibility of stopping testing early. This has only a very small impact on statistical power, as it only matters if the final *p* values are between 0.05 and 0.0294 and also the initial *p* values are above 0.0294. This technique is most helpful when the effect sizes of interest are difficult to estimate, allowing for an early stop if they are found to be large while also allowing for a large sample if they are found to be small. (There are also versions for three analyses, using an alpha of 0.0221; four analyses, 0.0182; five analyses, 0.0158; and so on.)

Power analysis was done via simulation. Optimal cue combination effects tend to be relatively large, often above *d* = 1.0 (Negen et al., 2019). We simulated the change from *d* = 0 in block 1 to *d* = 1.0 in block 3. This was done in 10,000 independent runs. Each run generated two columns of normally distributed bimodal VE advantages with a standard deviation of 1, a covariance of 0.5, and means of 0 and 1. Each run was then tested with a paired *t*-test on the first 20 and again with the whole simulated sample. This procedure found that the contrast between blocks had 85% power with the first 20 participants. With 40 participants, this increased to over 99%. Modifying the simulation slightly, we also found that a much more conservative change, from *d* = 0.25 to 0.75, had over 80% power with all 40 participants. For the regression for bias onto cue combination, we used G*Power. We found that a single slope with the same standardized beta as Experiment 1 (0.532 when using the same VE calculation as this experiment; see Coordination note) gives 85% power with just the first 20 participants and 99% with the full sample of 40. Although this could be decreased by issues such as collinearity, at the time of preregistration we still felt that this could be appropriate if such issues are small.

#### Apparatus

Testing took place in a specialized audiovisual lab used for presenting wide-angle stimuli with acoustically attenuating material on walls and ceilings. The room has an Optoma GT1080E projector (Optoma Corporation, New Taipei City, Taiwan), Grandview Fixed Frame acoustically transparent 7-foot screen (Grandview Screen, Guangzhou, China), and an acoustic setup of 17 VISATON SC5 speakers (VISATON GmbH, Nordrhein-Westfalen, Germany) driven by nine Lepy 2024A stereo power amplifiers (Lepy, Guangdong, China). This equipment is manipulated through a Serenity Quiet PC (Quiet PC Ltd., Malton, UK), with dual USB Focusrite 18i20 soundcards (Focusrite PLC, Wycombe, UK) and NVIDIA GTX 980 graphics card. The speakers are placed horizontally 10 cm behind the screen, at the height of a subject's head. The room is kept dark except for the projector. The participant sits at a small table in the center and enters responses with a mouse. The seating position places the participant at 140 cm from the center of the screen and aligned with the central speaker.

#### Stimuli

Every trial was audio, visual, or audiovisual. The targets could be at −32°, −26°, −20°, −15°, −10°, −5°, 0°, 5°, 10°, 15°, 20°, 26°, or 32° from straight ahead, aligning with the speaker array. (Please note that these positions differ slightly from those described in the preregistration. The speakers were reinstalled in the laboratory after the preregistration but before the data were collected.)

The audio cue was 300 ms of amplitude-modulated white noise. The audio cue was sent to the speakers with a sampling rate of 44,100 Hz and a bit depth of 24. The amplitude modulation function consisted of three 100-ms cosine cycles (shifted and inverted to begin at 0 and have the range of 0 to 1). White noise was multiplied by this modulation function to make the stimulus. In other words, the stimulus changed in amplitude from 0 to 1 and back to 0 over 100 ms three times in immediate succession.

The visual cue was a cloud of 64 red dots. They were evenly spread along the width of the screen and placed randomly along the height of the screen. They moved horizontally 1/5 of the way toward the target location over a 300-ms time period. (They stayed at the same height during this move.) The movement of the dots was the cue to the location of the target; specifically, accurately estimating the point at which the dots would converge, if the remaining 4/5 movement were completed, would recover the location of each target via the visual cue. We used this stimulus because it results in normally distributed responses (Negen et al., 2019) and can be tuned to match the audio cue in terms of expected response variance by increasing or decreasing the movement length. An approximate match in response variance across cues is important because that is the situation where cue combination effects are expected to be the strongest. During the stimulus presentation, the size of the dots was controlled by a sine wave with a half-period of 300 ms. This was done to create a sense of the audio and visual cues roughly corresponding in terms of their attack/fade timing, which may help participants integrate them. The maximum radius was 0.5% of the width of the screen.

#### Procedure

The procedure began with a set of instructions. The first screen began with the participant entering their age and gender identity. Next, a cartoon dog appeared with a speech bubble saying, “Hi, my name is Benji.” “Let's play hide and seek. I will hide somewhere down here.” Benji then appeared for 250 ms at the bottom of the screen in eight places in the following random order: −32°, 32°, three places randomly chosen from 0° to 32°, and three places chosen randomly from 0° to −32° for a period of 15 frames each. All further instructions were given via a speech bubble with the Benji character. The next instructions were: “For this game, we will use both our eyes and our ears.” “Sometimes I pull a bunch of red dots towards me. You can find me by seeing where all the dots are going.” A demonstration of the visual stimulus was then shown (target of 0°). Following this, similar instructions were given in the same way for the auditory only and audiovisual trials, each followed by a demonstration of the respective trial stimulus presented at the target location of 0°. The final instruction given was “Sometimes I pop back up to show you where I was but sometimes, I stay hidden.” “Ready, let's play!” following which the trials began.

All trials began with a small fixation cross in the center of the screen. The participant initiated the trial (i.e., removed the fixation and began the stimulus) by pressing the space bar. Participants responded by moving a computer mouse left/right, which moved a vertical bar on the screen. They then made a left click to select the location. This was, thus, a continuous judgment task rather than one with alternative forced choices. Because we collected continuous responses, we could determine biases by comparing responses to the same locations via the two different cues in single-cue conditions. We did not need additional conditions to directly compare two cues to each other, unlike in Experiment 1 which used a two-alternative forced choice method.

For block 0, which served as a practice block, there were nine trials: three audio trials, three visual trials, and three audiovisual trials. In each type of trial the targets were presented at −32°, 0°, or 32° presented in random order. No feedback was given during these trials.

Block 1 consisted of 156 trials. The participant was not made aware of the fact that the block had changed (which is consistent throughout the experiment). Of the trials presented, 52 were audio, 52 were visual, and 52 were audiovisual. Four of each trial type were presented at each of 13 possible targets (13 targets × 3 types × 4 repeats = 156). The trials were presented in random order, and no feedback was given.

Block 2 consisted of 26 trials: 13 audio and 13 visual (no audiovisual). Each trial type demonstrated each possible target once. The trials were presented in random order, and feedback was given for these trials. The feedback consisted of the Benji character appearing at the correct location along with a vertical thin blue line that was displayed on the screen. The purpose of block 2 was to give the participant some feedback useful for better calibrating their unisensory estimates, before testing for cue combination again. We chose 26 trials as a balance between giving them enough information to notice any major bias issues while also avoiding a very long gap between testing blocks (and thus increasing practice effects).

Block 3 followed the same parameters as block 1, but in block 3 feedback was given following the audio-only and the visual-only trials (feedback was not given for audiovisual trials). This was done to make sure the training in block 2 did not decay too much, which could weaken the effect of interest.

#### Data processing

Our aim was to measure VE and relative bias from the dataset. To do this, we assessed the VE for each participant, block, and trial type. This represents the amount of random noise in responses and is intended to be separate from systematic distortions. We also measured relative bias for each participant and block. This was intended to measure the size of systematic differences between the perceived place of visual and auditory cues that are physically aligned. Note that two cues can have zero relative bias if they are both biased away from the true physical location in the same way.

As a first step, to identify and exclude responses that were outliers, we selected all trials in blocks 1 and 3 and pooled them together. We calculated (signed) errors by subtracting the targets from the responses. The standard deviation of this entire pool was then calculated. Any error with an absolute value of more than 3 *SD* (17.18°) was considered an outlier as stated in the preregistration. Using this method, 172 trials were excluded from further analysis (1.41%).

For the next stage of data processing, we obtained intermediate variables in the following way. For each participant and trial type for each of the two larger blocks (1 and 3), we regressed responses onto targets and calculated three things: the slope of the fit line, the intercept of the fit line, and the standard deviation of the residuals. With 39 participants, this leaves us with an intermediary matrix of the following size: 39 participants × 3 trial types (audio, visual, audiovisual) × 2 blocks × 3 outcomes (slope, intercept, standard deviation).

The standard deviation was taken as a measure of VE, random noise as separate from systematic biases. For each participant, for each of the two larger blocks (1 and 3), the best single-cue VE was calculated by taking the minimum of the audio-only standard deviation and the visual-only standard deviation, producing a matrix of 39 participants × 2 blocks. The AV VE is a 39 participants × 2 blocks matrix of the standard deviations for the AV trials.

For each participant in each of the two larger blocks (1 and 3), the bimodal VE advantage was found by subtracting the audiovisual standard deviation from the best single-cue standard deviation. If there were no sampling error, a bimodal VE advantage above zero would indicate that they used the two cues together to reduce noise. The bimodal VE advantage is a matrix of 39 participants × 2 blocks.

For the relative bias index, we first calculated a deviation matrix. For each participant in blocks 1 and 3, for each target we calculated the value of target times slope plus intercept for the visual trials and also calculated the value of target times slope plus intercept for the audio trials (i.e., the average fitted response for visual-only and audio-only trials with each target). We then calculated the absolute difference between these two values. This resulted in a deviation matrix of size 39 participants × 2 blocks × 13 targets. The relative bias measure was averaged across targets within participants and within blocks. This resulted in a 39 participants × 2 blocks matrix of relative biases. If the reader imagines a fit line for the audio trials and a fit line for the visual trials, the relative bias is proportional to the area that separates the two lines.

After the preregistration, as part of a separate project, we considered whether it would be more appropriate to divide the standard deviation by the slope as a measure of VE. This would compensate for anyone showing a strong central tendency bias (e.g., pushing every response 20% of the way toward the center), which could decrease the standard deviation of residuals without actually improving the precision of perception. This procedure is described and justified extensively in another article (Aston, Negen, Nardini, & Beierholm, 2022). In addition to the preregistered analyses, we also calculated the main outcomes using this alternative method and found the same pattern of significance as reported below.

#### Analysis plan

The preregistered analysis had three parts. First, bimodal VE advantage in block 3 was tested against zero using a one-tailed *t*-test. This was used to confirm that some kind of cue combination was occurring in this task and population. Second, a paired one-tailed *t*-test was used to test for a larger bimodal VE advantage in block 3 than in block 1. This is the central outcome, suggesting that cue combination increases after the intervention. Third, there was a custom multiple regression test—specifically, an *F*-test using nested hypotheses. The idea of this multiple regression was to see if any increases in bimodal VE advantage might be linked specifically to decreases in relative internal bias in a way that is separable from key covariates such as block number. This third analysis was intended to show that the bimodal VE advantage and cue combination held a unique relation not explained by other covariates.

Because the logic of the third preregistered analysis is complex, we will explain it further here. The core idea is to see if there was a more specific link between relative bias and cue combination, something beyond just block number or pre-existing individual differences. Imagine a situation where the participants have a distribution of relative bias in the first block, some higher and some lower. They also have a distribution of how well the intervention works, some more and some less. If a change in cue combination behavior is not just a function of block number, but really related to relative bias, then we would expect more than just an average increase in cue combination across blocks. Those who started with low relative bias in block 1 and maintained this in block 3 should have strong cue combination behavior in both blocks. Those who started with a high relative bias and did not benefit much from the intervention should have weak cue combination behavior in both blocks. Those who started with high relative bias and decreased it greatly should have weak cue combination behavior in block 1 and strong cue combination behavior in block 3. This means that cue combination should be related to relative bias above and beyond block number. This also means that cue combination should be related to relative bias above and beyond a within-subjects correlation across blocks (i.e., it is not just that the same people had weak/strong cue combination in both blocks). With a large enough sample and effect, it should be possible to analyze this with a regression (*F*-test) comparing nested models. The null model predicted the bimodal VE advantage with an intercept, the block number, and a term that allowed the bimodal VE advantage in the two blocks to be correlated. The full model also allowed a linear relation between bias and cue combination.

For clarity, we provide the Wilkinson notation models here:

(8) $$\begin{array}{c} H_{0}:\;BVEA_{ij}∼1+I_{block3}+I_{block3}:Err_{i1}\; \end{array}$$

(9) $$\begin{array}{c} \;\;\;\;\;\;\;\;\;\;\;\;\;H_{1}:\;BVEA_{ij}∼1+I_{block3}+I_{block3}:Err_{i1}+B_{ij}\; \end{array}$$

where *BVEA_ij_* is the bimodal VE advantage for participant *i* and block *j*, *I_b__lock_*_3_ is an indicator for block 3 (i.e., 1 for block 3 and 0 otherwise), *Err_i_*_1_ is the difference between the predicted mean and observation for *BVEA_i_*_1_, and *B_ij_* is the internal relative bias for participant *i* and block *j*.

### Results

The results partially supported the main hypothesis. The following sections detail this through descriptive statistics, the preregistered analysis, and additional post hoc analysis.

#### Descriptive statistics

**Figure 6.**
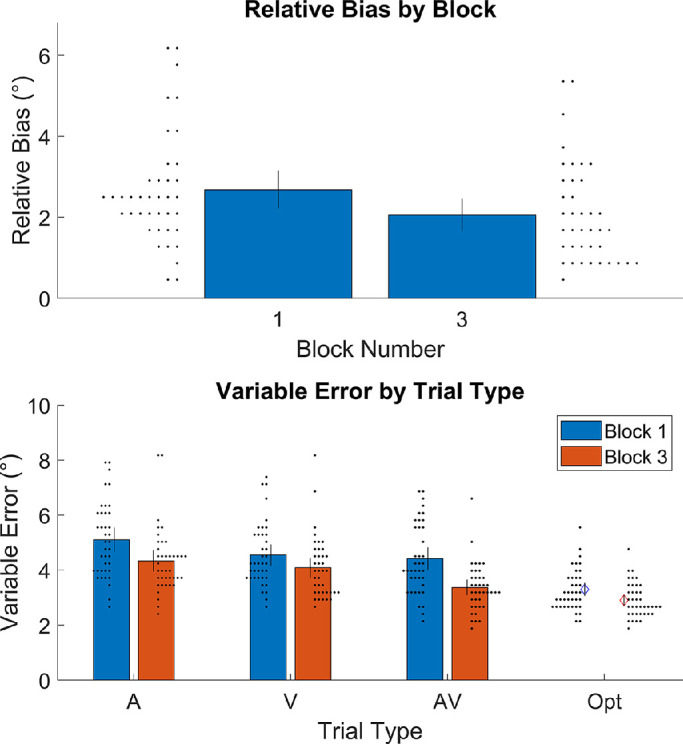
Observed measures of relative bias and VE. Bars are means. Black lines are 95% CIs. Small black dots are a histogram of the measurements associated with the nearest bar.

Figure 6 shows the means and individual data points for relative bias and VE. From block 1 to block 3, mean relative bias fell from 2.68° to 2.05°, *t*(34) = 3.13, *p* = 0.002, *d* = 0.53 (95% CI, 0.25°–1.00° decrease). This suggests that relative bias within the sample did decrease across blocks as intended. VE in the audio and visual trials were similar enough for present purposes, both falling between 4.0° and 5.5° on average. Average VE also improved (lowered) slightly from block 1 to block 3 for the audio trials (*d* = 0.670; 95% CI, 0.40°–1.14° decrease), visual trials (*d* = 0.546; 95% CI, 0.19°–0.74° decrease), and audiovisual trials (*d* = 0.912; 95% CI, 0.67°–1.42° decrease). Due to this, the optimal bimodal VE advantage (the largest theoretical decrease in VE with the optimal algorithm) also fell from block 1 to block 3, from 0.94° to 0.89°. This is convenient because it rules out the potential interpretation that people might show a larger cue combination effect in block 3 due to an increase in the possible benefit. The 71-year-old participant's data were not particularly notable; they had a mean relative bias of 5°, mean VE of 3.8°, and a mean bimodal VE advantage of 0.4°. These are all well within the ranges seen in the rest of the data.

#### Preregistered analysis

The three preregistered analyses partially support the main hypothesis. Following the logic of the Pocock boundary (described in Experiment 2), we tested all 40 participants and report the statistics of the full sample (39 after exclusion) here. The first analysis examined whether, overall, adults combine cues during this task. As stated in the preregistration, participants were excluded if their bimodal VE advantage was more than 1.96 *SD* from the mean (three exclusions). Bimodal VE advantage was significantly above zero in block 3, *t*(36) = 5.07, *p* < 0.001, *d* = 0.83, confirming that, overall, participants do combine cues.

**Figure 7.**
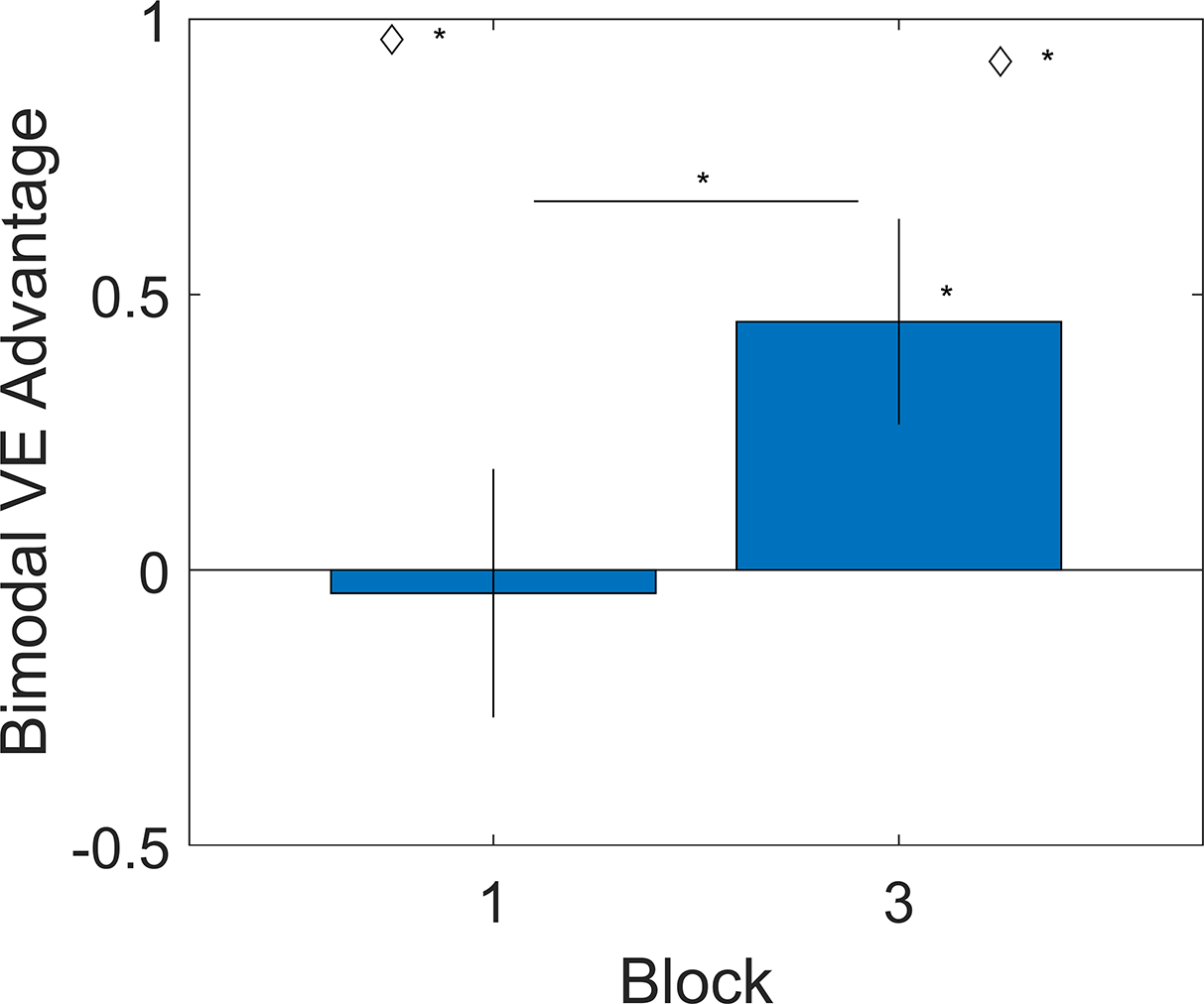
Bimodal VE advantage as a function of block number in Experiment 2. The bimodal VE advantage is the reduction in VE that participants show when they have both cues versus their best single cue. This is expressed in degrees. The difference between blocks (i.e., pre-training and post-training) was statistically significant. The advantage was significantly non-zero in block 3 (i.e., post-training). Diamonds indicate the optimal advantage that was possible. The advantage was significantly suboptimal in both blocks. This is consistent with the main hypothesis.

The second analysis is the most critical one, examining whether cue combination behavior increased after the training (Figure 7). There were four outlier exclusions. The bimodal VE advantage was larger in block 3 than in block 1, *t*(34) = 3.45, *p* = 0.001, *d* = 0.58, confirming that cue combination behavior increased. (Please note that this is despite an average decrease in the potential benefits; see Descriptive statistics.)

For the third preregistered analysis, the model comparison was not significant, *F*(1, 70) = 0.68, *p* = 0.411. Two participants were excluded as outliers. This means that we failed to find significant evidence for a specific relation between relative bias and cue combination that goes beyond block number and the possibility of an across-block within-subjects correlation.

In summary, the preregistered analyses did find group-level evidence for the main hypothesis, with the bimodal VE advantage increasing after training (with a decrease in relative bias, as well), but no evidence for individual-level correlation was found, with no significant correlation between bias and bimodal VE advantage when controlling for block number.

In this case, we decided that it would be of benefit to examine this result further with an unregistered post hoc model analysis. The results above show a change across blocks as expected, but no particular evidence that the change in relative bias is related to the change in cue combination. It is not yet clear if this is due to a lack of relation between bias and combination, or if this might just be due to the difficulty of finding a significant regression result when there are multiple collinear predictors. We filled this gap with a trial-level post hoc analysis that used only data in block 1. The following section details this.

#### Post hoc target-level modeling in block 1

**Figure 8.**
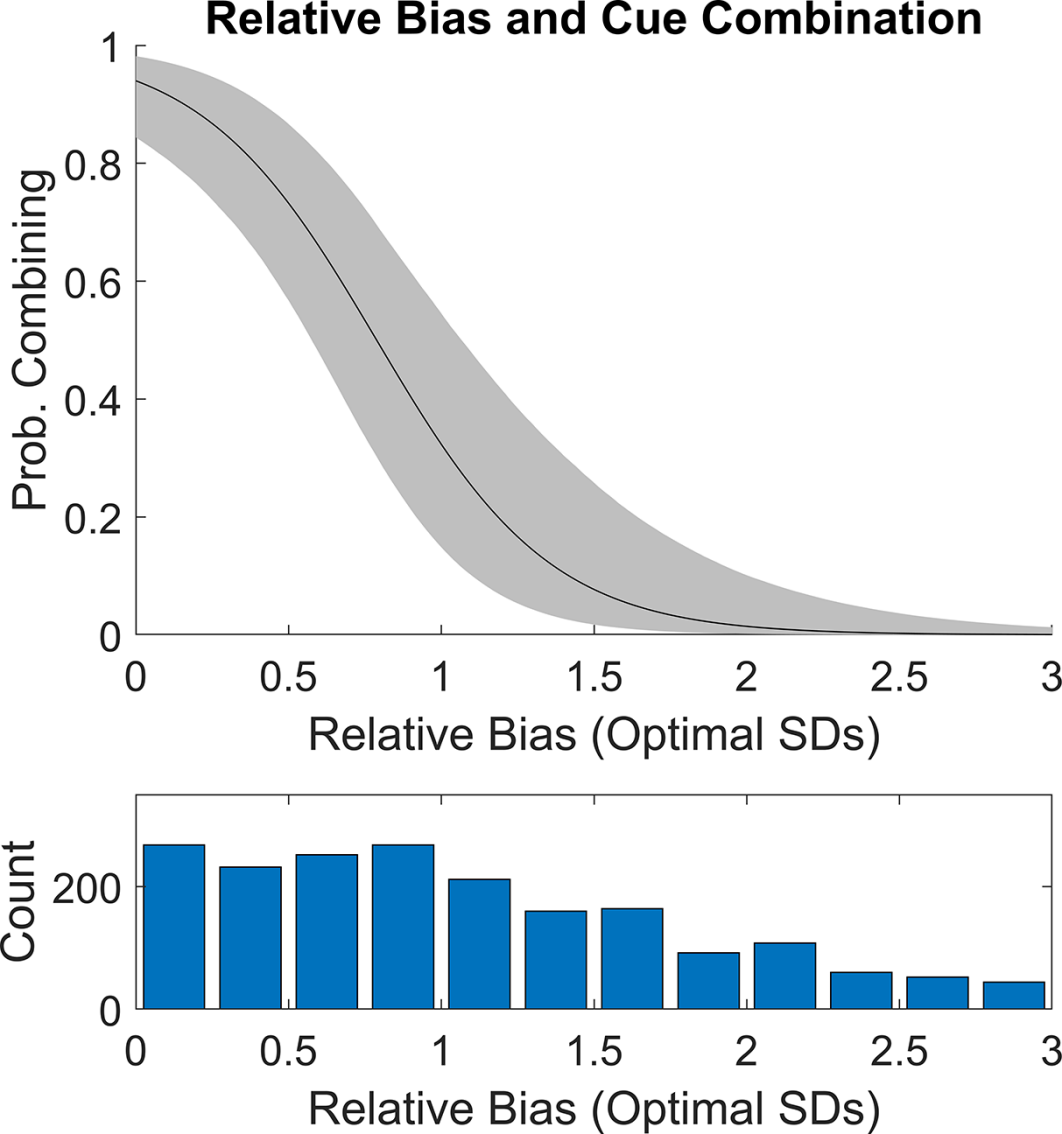
Inferred probability of combining cues on an audiovisual trial during block 1 in Experiment 2 as a function of relative bias. In the top panel, the black line is calculated using the mean posterior values of the relevant parameters (µ*_C_*, β*_C_*). The gray patch is the 95% credible interval. The inferred probability of combining the two cues drops as the relative bias increases. In the bottom panel is a histogram that shows how many trials had different levels of relative bias. This shows that there was some appreciable amount of data to constrain the curve above through the displayed range.

Modeling results suggested that the target-to-target variation in internal relative bias did predict cue combination behavior in block 1. We will detail the analysis method below. In summary, we took advantage of the fact that internal relative bias varied by target within the same participant for a more detailed analysis of the relation to cue combination behavior. The central result is shown in Figure 8. In short, we estimate that participants combine cues on over 85% of trials when relative bias is zero. When relative bias reaches two units (with a single unit being the standard deviation that could be achieved optimally), participants combine cues on fewer than 10% of trials.

Our broad approach was to fit a Bayesian cognitive model and examine the posterior credible intervals (analogous to confidence intervals). We chose to look exclusively at block 1 so that the training could not be a potential third variable that might explain any discovered relation. The model allows each participant to have a separate intercept, slope, and standard deviation for each single cue. This allows for each participant and target to have a different relative bias. For example, a given participant might have a relative bias of 0° for the center target but may have a relative bias of 2° out at the −26 and +26 targets. This would occur if the audio and visual intercept matched (e.g., +1.5° and +1.5°) but their slopes were offset by 1/13 degrees per degree (e.g., 1.038° and 0.962° per degree). We then modeled the probability of combining cues as a function of the amount of relative bias.

The single-cue trials were modeled in a way that resembled simple linear regression; each response is modeled as the target times a slope plus an intercept plus some noise. The probability of a response *x* for an audio trial with target *t* is

(10) $$\begin{array}{c} \;\;\;\;\;\;\;\;\;\;\;\;\varphi \left(x|\mu =t\beta_{jA}+I_{jA},\sigma =S_{jA}\right)\;\times \;0.99+\;\frac{0.01}{90}\;\;\; \end{array}$$

where φ is the normal probability density function, β*_jA_* is the slope, *I_jA_* is the intercept, and *S_j__A_* is the standard deviation of responses around the mean. The subscript *j* indicates that a variable is separate for each participant. The fraction term on the right is a lapse mechanism, allowing for 1% of responses to be uniform random draws. The 0.99 factor is present because the participant is modeled as having a 99% chance of a non-lapse response. The visual trials were modeled in the same way but with independent parameters β*_jV_*_,_ *I_jV_*, and *S_jV_*.

The audiovisual trials were modeled as a mixture of four different distributions. These represent the use of the audio cue alone, using the visual cue alone, lapsing and guessing randomly, and using both cues in combination with optimal weights. These were

(11) $$\begin{array}{c} P_{A}=\varphi \left(x|\mu =t\beta_{jA}+I_{jA},\sigma =S_{jA}\right)\; \end{array}$$

(12) $$\begin{array}{c} P_{V}=\varphi \left(x|\mu =t\beta_{jV}+I_{jV},\sigma =S_{jV}\right)\; \end{array}$$

(13) $$\begin{array}{c} P_{L}=1/90\; \end{array}$$

(14) $$\begin{array}{ccc} \;P_{AV} & = & \varphi \left(x|\mu =t\beta_{jAV}+I_{jAV},\sigma =\beta_{jAV}\right.\\ & & \;\times \left.{\left({\left(S_{jA}/\beta_{jA}\right)}^{-2}+{\left(S_{jV}/\beta_{jV}\right)}^{-2}\right)}^{-1/2}\right)\; \end{array}$$

The *P_AV_* term is again like a linear regression, with each response being modeled as the target times the slope plus an intercept plus some noise. The important thing is that the standard deviation is the optimal standard deviation that can be achieved with the limitations of the two cues. The terms with β in the standard deviation expression are present because an overall slope can be used to make the standard deviation of responses more/less than the standard deviation of the underlying perception (Aston et al., 2021). The divisions and multiplications in the equation correct for this.

The final probability of an observed audio-visual response is

(15) $$\begin{array}{ccc} & & 0.01P_{L}+0.99\left(CP_{AV}+\left(1-C\right)\right.\\ & & \left.A_{j}P_{A}+\left(1-C\right)\left(1-A_{j}\right)P_{V}\right)\; \end{array}$$

where *C* is the probability of combining cues and *A* is the probability of using the audio cue alone when not combining cues. The 0.01 and 0.99 factors indicate that there was a 1% chance of a lapse response and a 99% chance of a non-lapse response. *A* was independent for each participant. *C* was modeled as

(16) $$\begin{array}{c} C=\frac{e^{\mu_{C}+R\beta_{C}}}{1+e^{\mu_{C}+R\beta_{C}}}\; \end{array}$$

where *R* is the relative bias for that target and participant, µ*_C_* is a global parameter for the rate of cue combination, and β*_C_* is a global parameter for the effect of relative bias on cue combination behavior. To be clear, *C* was different for every target and participant, but µ*_C_* and β*_C_* were the same for all trials.

For computational convenience, we sampled the logarithm of σ rather than σ itself. Most parameters were not given explicit priors, with the following exceptions:

(17) $$\begin{array}{c} \beta_{jAV}∼N\left(\mu =1,\;\sigma =0.25\right)\; \end{array}$$

(18) $$\begin{array}{c} I_{jAV}∼N\left(\mu =0,\;\sigma =1\right)\; \end{array}$$

(19) $$\begin{array}{c} \beta_{C}∼N\left(\mu =0,\;\sigma =1\right)\; \end{array}$$

(20) $$\begin{array}{c} \mu_{C}∼N\left(\mu =0,\;\sigma =10\right)\; \end{array}$$

The remaining variables were well constrained by the data.

Note that these priors do not favor the hypothesized relation between relative bias and cue combination. We simulated two datasets where the relation between relative bias and cue combination was flat (β*_C_* = 0) or negative (β*_C_* = −1) and recovered appropriate inference.

Sampling was performed with slice-sampling in MATLAB. Six independent chains were run with 5250 samples each. The first 250 were discarded as burn-in. The $\hat{R}$ scale reduction factors were between 0.999 and 1.001 for all parameters, which points towards an adequate number of samples. To avoid issues with edge effects, the data from the most extreme targets (−32 and +32) were not used. The results shown in Figure 8, suggesting that target-to-target variation in internal relative bias strongly predicts cue combination behavior, were calculated as the inner 95% percentile of sample estimates for the displayed quantities.

### Discussion

On balance, we interpret the results from Experiment 2 as supporting our prediction. Preregistered analyses provided evidence that, after additional experience with feedback designed to lower relative internal bias, bias was indeed lowered, and there was also an average increase in cue combination behavior. This is consistent with reduced biases leading to improved cue combination. However, we did not find a significant participant-level effect of relative bias on cue combination behavior that went beyond trial number and the possibility of a within-subjects across-blocks correlation in cue combination. It is not necessarily clear if this is because the effect does not exist or because it is so difficult to find it in a regression with multiple collinear predictors. We also showed post hoc evidence that, before the intervention, the variation in cue combination behavior is indeed related to the variation in internal relative bias, as it was in Experiment 1. This post hoc finding at least suggests that some relation between relative bias and cue combination was present, making it a plausible mechanism to explain why cue combination increased after the training.

## General discussion

We interpret the findings from both experiments as strong, if somewhat preliminary, reasons to believe that internal relative bias can disrupt cue combination behavior. Using an individual differences approach in Experiment 1, we found a predicted cross-sectional correlation between internal relative bias and cue combination behavior during 3D perception in 7- to 10-year-olds. Using a training approach in Experiment 2, we found a predicted increase in cue combination behavior after an intervention designed to decrease internal relative bias during audiovisual localization in adults. A post hoc modeling analysis of the pre-training data in Experiment 2 also found a cross-sectional relationship in the variation in naturally occurring biases and cue combination in this different population and task.

Each method has necessary limitations, but the two approaches are mutually supportive. The individual difference findings in Experiment 1 and the post hoc analysis of Experiment 2 could be due to a third variable, but the main within-subjects finding in Experiment 2 cannot be due to any pre-existing individual differences. The main finding in Experiment 2 could be a practice effect, but the finding in Experiment 1 and post hoc analysis of Experiment 2 cannot be. We have also found some reasons to rule out some obvious alternative explanations. The correlation in Experiment 1 still held after controlling for age, so that is unlikely to be a relevant third variable. In Experiment 2, the scope for the maximum size of the cue combination effect did not increase after the intervention. If we suppose the main results of Experiment 2 are due to practice, that does not explain the post hoc finding that target-to-target variation in internal relative bias is related to cue combination behavior. The major weakness in the evidence is that we did not find the predicted participant-level correlation between internal bias changes and cue combination behavior changes during Experiment 2, although this could be due to issues with statistical power and collinear predictors. On balance, these data do seem to support the main hypothesis.

We suggest that these results are strong enough to provide a useful avenue of further investigation when researchers fail to find a cue combination effect (or near-optimal cue combination effect) in other situations. A typical forced-choice two-alternative design does not generally allow for the biases across cues to be measured. However, it can be adapted readily to provide such a measurement with cross-cue trials. This could identify any important subsets of the data/population where (near-optimal) cue combination is occurring and provide a potential mechanism for the variation. Alternatively, if the internal relative bias is measured thoroughly and found to be negligible, it would allow a greater level of certainty that the result is not due to issues with the calibration of single-cue perception. Further investigation into relative bias could therefore clarify and deepen the results in the wider field.

These results might also help answer a long-standing question: Why do children in middle and late childhood generally fail to combine cues? The key may simply be that cues are not combined by perceptual processes when internal relative bias is too high, and many people under 10 years of age have high internal relative bias. This would explain the major behavioral findings in this area. In most studies, children under 10 years old do not combine cues (Burr & Gori, 2011). In a previous study, we found that children who are 7 to 10 years old can combine cues when they are given a few minutes of accurate feedback on single-cue trials (Negen et al., 2019). In Experiment 1, we found that a subset of children who have below-median internal relative bias also combine cues without any feedback while replicating the typical result showing that the full sample shows no significant cue combination effect. All of this can be potentially explained at the mechanism level by positing that high levels of internal relative bias disrupt cue combination behavior.

These results may point toward an explanation for inconsistent findings in previous reports, as well. Although one cornerstone study found near-optimal cue combination for audiovisual judgments of horizontal location (Alais & Burr, 2004), a number of related studies have failed to find a similar effect (Arnold et al., 2019; Battaglia et al., 2003; Burr et al., 2009; Garcia et al., 2017; Maiworm & Röder, 2011). It is possible that this is due to differences in relative internal bias in these slightly different tasks and samples.

The results presented here may seem somewhat at odds with a previous report that found cue combination on a task that showed measurable internal relative bias (Scarfe & Hibbard, 2011). We note that our prediction was not that having *any* measurable bias would *eliminate* combination—only that having more bias would lead to less effective combination. Indeed, in Experiment 1, the subset of combiners had, on average, a substantial measurable bias (see Figure 2). Nevertheless, it is important to consider this earlier study a rare example of an approach to measure both naturally occurring biases and cue combination. One possible explanation for why combination was seen in the Scarfe and Hibbard task is that internal relative bias levels were still too low to disrupt cue combination, as in our Experiment 1 combiners. Their simulations suggest that combining the two cues, even though this introduces bias into the estimate, still has a net benefit in terms of mean squared error. Another thing to point out is that the authors were not specifically investigating individual differences or correlation but rather were attempting to describe normative behavior. It is therefore possible that a correlation analysis performed on data acquired from a similar study with a larger sample would produce results akin to those presented in this report.

To be clear, nothing here necessarily suggests that internal relative bias is the sole reason why children under 10 years of age do not combine cues, nor does it suggest that decreasing internal relative bias is the sole reason why an intervention might increase cue combination behavior. For example, in order to set weights, a person must have reasonable estimates of their own internal noise. It is possible that the same participant may have both high internal relative biases and inaccurate internal noise estimates. These might both have to be overcome to begin gaining precision from multiple cues.

In summary, available evidence points toward the idea that high levels of internal relative bias disrupt cue combination. This may be a key to understanding why developing children fail to combine cues and why adult audiovisual cue combination is inconsistent. Further studies should investigate whether this pattern is also present in a wider variety of samples and tasks. Researchers who fail to find an expected (near-optimal) cue combination effect should consider collecting additional measures to test whether this could be explained by high levels of relative internal bias.

## Supplementary Material

Supplement 1
